# Programmable definition of nanogap electronic devices using self-inhibited reagent depletion

**DOI:** 10.1038/ncomms7940

**Published:** 2015-04-27

**Authors:** Brian Lam, Wendi Zhou, Shana O. Kelley, Edward H. Sargent

**Affiliations:** 1 Department of Pharmaceutical Sciences, Leslie Dan Faculty of Pharmacy, University of Toronto, Toronto, Ontario M5S 3M2, Canada; 2 Department of Electrical and Computer Engineering, Faculty of Engineering, University of Toronto, Toronto, Ontario M5S 3M2, Canada; 3 Department of Chemistry, Faculty of Arts and Sciences, University of Toronto, Toronto, Ontario M5S 3M2, Canada; 4 Institute for Biomaterials and Biomedical Engineering, University of Toronto, Toronto, Ontario M5S 3M2, Canada; 5 Department of Biochemistry, Faculty of Medicine, University of Toronto, Toronto, Ontario M5S 3M2, Canada

## Abstract

Electrodes exhibiting controlled nanoscale separations are required in devices for light detection, semiconductor electronics and medical diagnostics. Here we use low-cost lithography to define micron-separated electrodes, which we downscale to create three-dimensional electrodes separated by nanoscale gaps. Only by devising a new strategy, which we term electrochemical self-inhibited reagent depletion, were we able to produce a robust self-limiting nanogap manufacturing technology. We investigate the method using experiment and simulation and find that, when electrodeposition is carried out using micron-spaced electrodes simultaneously poised at the same potential, these exhibit self-inhibited reagent depletion, leading to defined and robust nanogaps. Particularly remarkable is the formation of fractal electrodes that exhibit interpenetrating jagged elements that consistently avoid electrical contact. We showcase the new technology by fabricating photodetectors with responsivities (A/W) that are one hundred times higher than previously reported photodetectors operating at the same low (1–3 V) voltages. The new strategy adds to the nanofabrication toolkit method that unites top–down template definition with bottom–up three-dimensional nanoscale features.

## Abstract (web summary)

The miniaturization of electronic circuitry requires the reproducible fabrication of electrodes with nanoscale separations. Here, the authors present a robust manufacturing technology that enables programmably placed nanoscale three-dimensional nanogaps on an integrated circuit.

## Graphical abstract

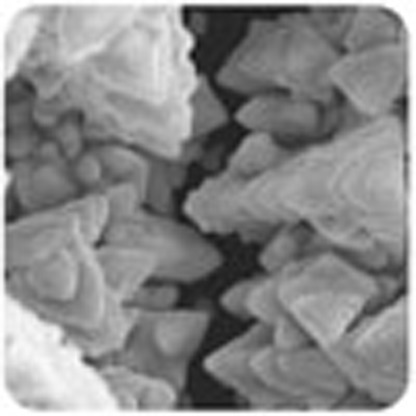

A half century ago, the self-aligned gate technique^1^, combined with progress in micron-scaled lithography^2^, enabled robust fabrication of integrated electronic circuits using combinations of field-effect transistors. Ensuing advances in nanoscale feature definition have enabled the latest electronic devices as well as novel biosensors^3^,^4^,^5^,^6^,^7^,^8^,^9^, catalysts^10^,^11^ and photovoltaic devices^12^,^13^,^14^,^15^. Various methods have been developed to fabricate nanoscale features with nanometre separations, including block copolymer lithography^16^,^17^,^18^, galvanic displacement^19^,^20^, interference lithography^21^,^22^ and nanosphere-patterned etching^23^,^24^. These methods are generally inexpensive and scalable; however, they typically lack top–down control required to fabricate addressable nanoscale-separated electrodes.

The definition of addressable isolated electrodes at the nanoscale^25^ can be performed with serial direct-write methods including dip-pen^26^,^27^ and electron beam lithography^28^,^29^. These offer impressive critical dimensions with precise top–down control that, however, can be difficult to scale. Electromigration^30^,^31^ can provide a fast and simple fabrication route to nanogap electrodes, but sizes are difficult to control reliably. Nanoimprint^32^,^33^ lithography offers a scalable and parallel route for fabrication, but is limited by fabrication of imprint masters and physical contact. Electrochemical^34^,^35^,^36^ methods rely on predefined electrodes that are electroplated to reach nanoscale separation distances, but rely on electron beam lithography and/or sensitive feedback systems. In sum, it remains challenging to fabricate, using previously established methods, controllably placed individually addressable nanogap electrodes.

Here we show a simple and inexpensive electrochemical self-inhibiting approach to fabricate robust and scalable nanoscale separations between laterally disposed electrodes. We hypothesized that, under certain electrodeposition conditions, a pair of electrodes undergoing simultaneous electroplating at the same potential could exhibit self-inhibition in their growth. Specifically, depletion of reagents in the narrow region between electroplating electrodes could produce the desired self-limiting mechanism. We termed this prospective mechanism, which would require verification via both experiment and modelling, self-inhibited reagent depletion (SIRD).

## Results

### SIRD method overview

**Figure 1:**
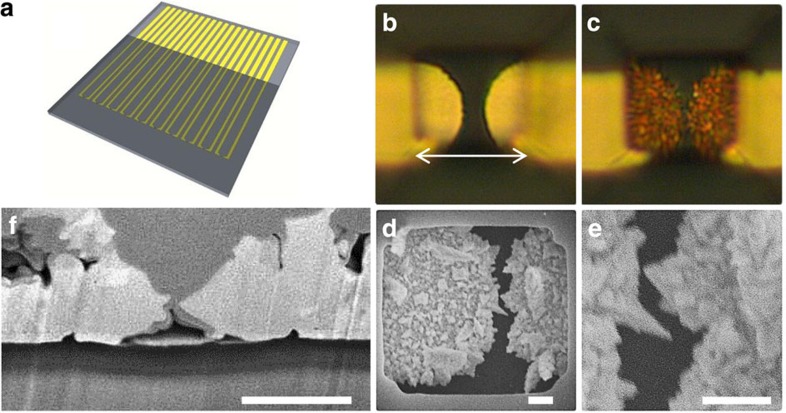
Self-inhibiting reagent depletion electroplating platform. (**a**) Lithographic template fabrication. Electrical leads with a 2 μm separation distance are passivated with a layer of SU-8 photoresist. (**b**) Ten-μm apertures (denoted with an arrow) are imaged into the SU-8 passivation layer above the 2 μm separation. (**c**) Imaging after SIRD electroplating electrodes in parallel with 2 mM HAuCl_4_+50 mM HCl at 200 mV for 3 min. (**d**,**e**) SEM top–down imaging after SIRD electroplating. (**f**) Focused ion beam cross-sectional imaging after SIRD electroplating. (Scale bar, 1 μm).

We began by forming a scaled-up version of the intended chip architecture, a template that would be easily realized using standard photolithographic techniques. These chips consisted of pairs of independently addressed coplanar electrodes (Fig. 1a) separated by 2 μm gaps at the closest point between each electrode pair. The surface of the chip was passivated using a layer of negative photoresist (SU-8) into which 10-μm-diameter square apertures exposed the narrow separated region (Fig. 1b). All electrodeposition was carried out with electrode pairs simultaneously held at the same potential.

Our initial tests were performed with a gold electroplating solution consisting of 2 mM HAuCl_4_ and 50 mM HCl as a supporting electrolyte. Electrodes were electroplated simultaneously at a potential of 200 mV versus Ag/AgCl for 5 min (Fig. 1c). Surprisingly, electrical shorts between electroplated pairs of electrodes did not form, as confirmed through simple electrical short testing. Using scanning electron microscopy (SEM) analysis, we observe the spacing between electrodes at the nearest point to be ∼50 nm (Fig. 1d,e). Since electroplated electrodes are non-planar in nature, we also performed focused ion beam cross-sectional imaging (Fig. 1f) to confirm that the nearest point was ∼50 nm.

### SIRD method characterization

**Figure 2:**
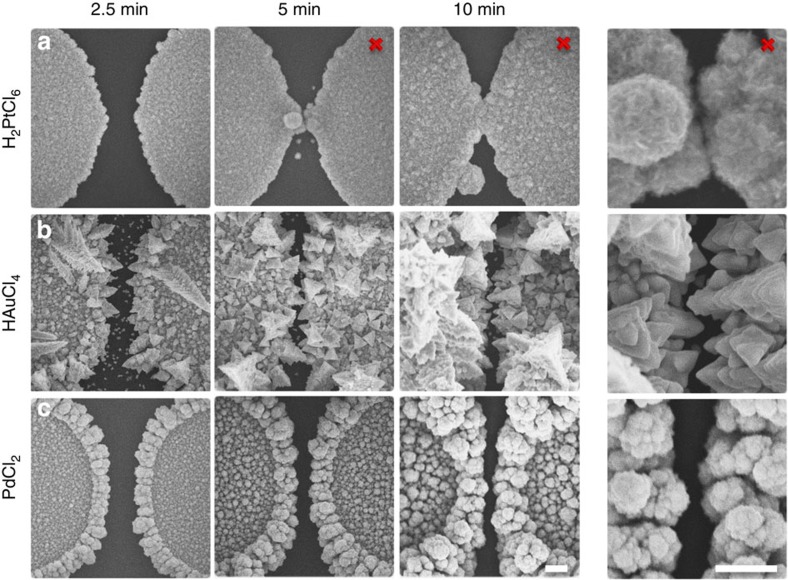
Comparison of SIRD electroplating parameters. (**a**–**c**) Time course of SIRD electrodes electroplated with different metal salts at a concentration of 2 mM and supporting electrolyte of 50 mM HCl. (**a**) Electroplating SIRD electrodes using a species with slow-plating kinetics (H_2_PtCl_6_). (**b**) Electroplating SIRD electrodes a species with fast-plating kinetics (HAuCl_4_). (**c**) Electroplating SIRD electrodes with intermediate plating kinetics (PdCl_2_). Images marked with a cross represent shorted electrodes. (Scale bar, 1 μm).

To investigate the versatility of SIRD, we electroplated devices using a number of different metal salts (Fig. 2). For comparison, each metal salt is kept at a 2 mM concentration with 50 mM of HCl as supporting electrolyte and the structures formed are compared across the same plating periods. We tested H_2_PtCl_6_ (Fig. 2a) to investigate whether SIRD would occur with a metal with slower-plating kinetics. We found that electroplating devices with platinum readily caused electrically shorted connections to be formed even with plating periods <5 min. We explain this by noting that H_2_PtCl_6_ has the tendency to deposit isotropically.

**Figure 3:**
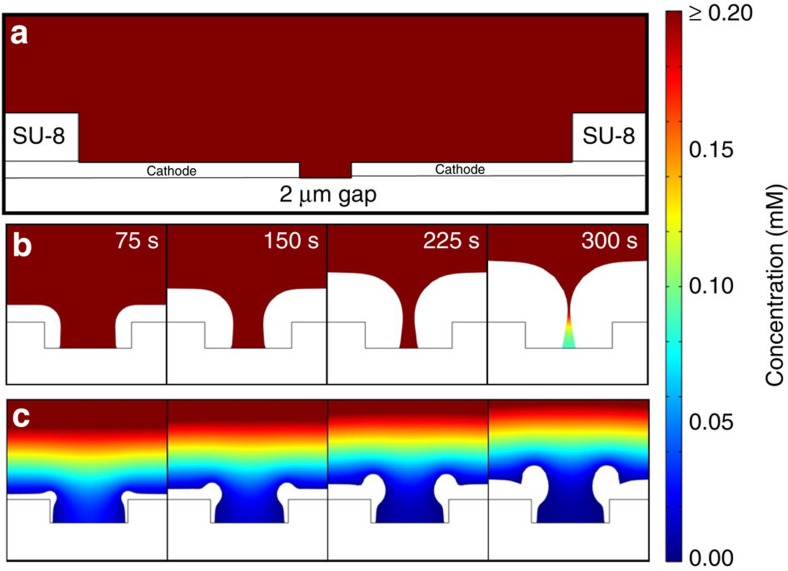
Simulation of SIRD electroplating phenomena. (**a**) COMSOL SIRD device configuration and initial conditions. Simulations show (**b**) isotropic electroplating of species with slow-plating kinetics and (**c**) anisotropic electroplating of species with fast-plating kinetics. (Heat map represents plating species concentration).

**Figure 4:**
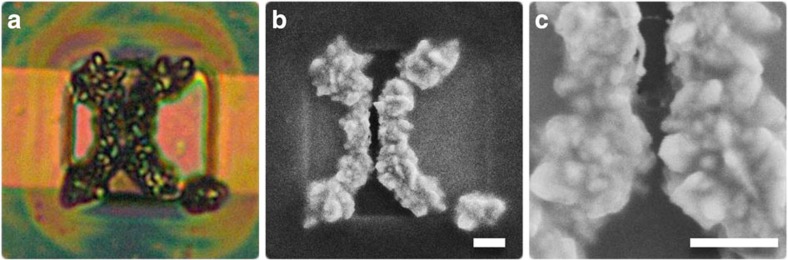
SIRD solution-processed photoconductors. PbS colloidal quantum dots are dip coated on surface of SIRD devices and infiltrate narrow region creating nanoscale photoconductor junctions. (**a**) Image of SIRD photoconductors with corresponding SEM images. (**b**,**c**) SEM images. (Scale bar, 1 μm).

**Figure 5:**
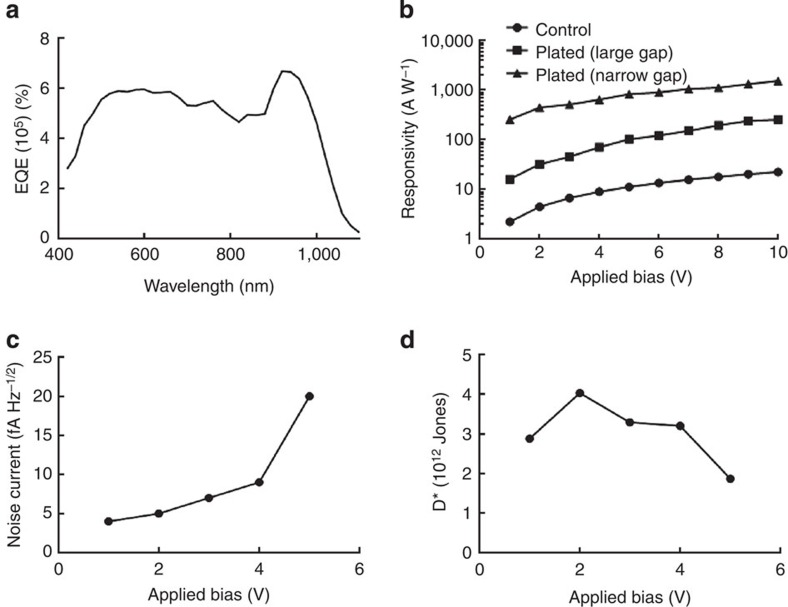
SIRD photoconductor characterization. (**a**) Spectral external quantum efficiency. (**b**) Responsivity with respect to applied bias. (**c**) Noise current with respect to applied bias. (**d**) Detectivity with respect to applied bias.

We reexamined HAuCl_4_ in greater detail to explore whether SIRD was in fact the mechanism underlying the formation of nanoscale gaps (Fig. 2b). Since electroplating is anisotropic, it can be difficult to image comprehensively via SEM (Fig. 2b—5 min) if electrodes are shorted; however, a simple electrical short test is also performed as confirmation. Using both SEM analysis and electrical short testing, we found that electrical shorts do not form even when electrodeposition proceeds for 10 min. This finding is particularly remarkable since HAuCl_4_ electroplates random fractal structures with jagged elements greater than the base electrode separation distance.

Next, we tested PdCl_2_ as a species that is known to have faster-plating kinetic (Fig. 2c). Using both electrical short testing and SEM imaging, we found that shorts do not form even after >10 min of electrodeposition. Electroplating with PdCl_2_ is also anisotropic, however, not as fractal as HAuCl_4_, resulting larger electrode separations >100 nm.

Interestingly, plating with H_2_PtCl_6_ causes electrical shorts to form even for shorter-plating times (<5 min) than faster-plating moieties at longer-plating periods (>10 min). We hypothesize that this is due to slow electroplating kinetics of plating reagents, where slow electron-transfer kinetics causes reagents to be consumed slowly near electrodes. Since plating reagents are consumed slowly they can be readily replenished, even in narrow separation regions. This causes electrodes to plate isotropically and readily form shorts.

Electroplating with HAuCl_4_, we form electrode pairs that remain isolated for longer-plating periods (>10 min) and have a separation distance of 50 nm or less. Due to the three-dimensional nature of HAuCl_4_ electroplating, SEM imaging can show plated electrodes that overlap (Fig. 2b—5 min). Surprisingly, these overlapping electrodes remain electrically isolated. This indicates that HAuCl_4_ surrounding plating electrodes is replenished at a slower rate than that at which it is consumed, especially in the narrowing regions. Since the plating reagent is depleted in the narrow region, this prevents growth to a physical connection and thus against formation of electrical shorts. Electroplating with PdCl_2_, we can plate electrode pairs that remain isolated with larger separation distances (∼100 nm). Again, this indicates that PdCl_2_ is consumed at a much faster rate than it is being replenished resulting in electrodes that remain indefinitely isolated with larger separation distances.

### SIRD electrochemical modelling

We turned to physical modelling to investigate in further detail the potential mechanisms at work. To model reagent consumption during electroplating, we employed COMSOL electrodeposition analysis (Fig. 3) in self-consistent two-dimensional finite-element time-domain simulations. A cross-sectional geometry matching cross-sections of our devices (Fig. 3a) was utilized to build physically representative simulations. Two electrodes separated by 2 μm below a passivation layer were used as cathodes and the corresponding anode is placed far away in the simulated electrochemical cell. The concentration of metal salt solution was specified to 2 mM to match the experimental conditions. The exchange current density of plating reagents indicates the rate of electron transfer between the electrode and plating reagent. The diffusion coefficient was estimated from measured steady-state currents (Supplementary Fig. 1). Slow Pt- and fast Pd-plating conditions were simulated with a species charge of 4 and 2, respectively, with the same electroplating overpotential. A wide range of exchange current densities were modelled and electroplating morphologies from exchange current densities of 5 A m^−2^ (Fig. 3b) and 150 A m^−2^ (Fig. 3c) matched experimental plating of Pt and Pd, respectively, at 5 min.

Simulation of the slower deposition rates reproduce isotropic growth seen in experiments, while faster rates reproduce the observed anisotropic growth. The simulations under slow-plating conditions confirm that electroplating kinetics occur at a sufficiently low rate such that the supply of plating species in the narrow region between electrodes is able to meet the need for reagent resupply in the steady state. This is evident in the higher concentration of plating species in the narrowing region (Fig. 3b) compared with the faster-plating simulation (Fig. 3c). In addition, electroplating kinetics are slow enough that electric field gradients at the edge of electrodes in the narrow region do not perturb the isotropic plating. Fast electroplating simulations reveal an insufficient supply of plating species in the narrowing region (Fig. 3c), evidenced by the negligible concentration of plating species in the narrow region.

### Solution-processed photoconductors

We sought to feature a useful application of SIRD in devices of practical interest. The field of low-cost solution-processed photodetectors is of interest in both ambient sensing and in the realization of highly multiplexed image sensors for camera systems. Until recently, solution-processed sensors had trailed in performance compared with solid-state crystalline semiconductor devices. However, recent advances in the semiconducting active materials have revealed that such photodetectors can, in principle, approach the performance of crystalline solid-state devices^37^.

Low cost of manufacture is a central principle in solution-processed devices. In lateral solution-processed photodetectors, this has led to a preponderance of studies focusing on multi-micrometre-spaced electrodes. This has the direct consequence that, to achieve a lateral electric field of amplitude sufficient to obtain efficient charge extractions, high biases in the range 50–100 V have been required. We took the view that solving the high-voltage-bias problem by producing low-cost nano-spaced electrode devices would directly showcase the appeal of SIRD.

The SIRD devices we fabricated for solution-processed photoconductor studies are shown in Fig. 4. A thin oxide coating of ∼50 nm was added to SIRD devices as a passivation layer to reduce background currents. The thin oxide layer caused electroplating to occur exclusively at the edges of the electrodes, where oxide breakdown and edge field effects allowed electrodeposition, whereas it did not occur elsewhere on the otherwise well-passivated structures (Fig. 4). SIRD oxide devices were fabricated in a similar manner to the SIRD mechanistic studies (Fig. 1) and used simultaneous electrodeposition in 2 mM HAuCl_4_ and 50 mM of HCl at 200 mV versus Ag/AgCl for 5 min. PbS colloidal quantum dots (CQDs) were synthesized as in previous reports^38^. SIRD devices were dip coated in CQDs in octane similar to a previously reported method^39^. The resultant CQD SIRD photodetector devices showed uniform coverage and response across electrodes.

Photodetector performance is evaluated through the figures of merit responsivity (*R*, in A W^−1^), noise-equivalent power (NEP, in W Hz^−1/2^), noise current (*i*_n_, in A Hz^−1/2^) and specific detectivity (D*, in Jones (cm W^−1^ Hz^−1/2^)). Responsivity quantifies the electrical current per optical power incident. The NEP is the minimum optical power impinging on the detector's active area that can be discerned above the noise level. The specific detectivity allows comparison among devices having different areas and bandwidths:


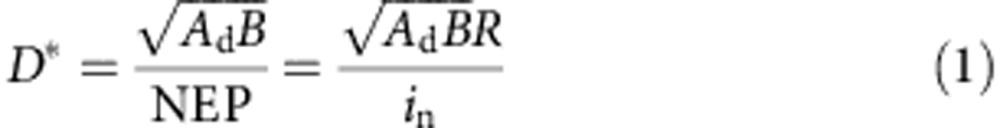


where *A*_d_ is the detector area, *B* the electrical bandwidth and *i*_n_ the noise current.

We present the optoelectronic performance of the SIRD photodetectors in Fig. 5, whose qualitative behaviour is consistent with previously reported works^39^. Figure 5a shows the external quantum efficiency (EQE) spectrum: a peak at 920 nm is seen that corresponds to the first excitonic peak of PbS nanocrystals, very close to the 929 nm observed for this set of nanoparticles in the solution phase following their synthesis. The SIRD photodetectors are two orders of magnitude more responsive at 450 nm wavelength at a given voltage bias (Fig. 5b), compared with unplated controls, as a result of higher electric field. Similarly, compared with plated devices with gap sizes of about 500 nm, the devices with narrow gaps are one order of magnitude more responsive. The unplated controls closely reproduce the best previously reported CQD performance^39^.

We also characterized the devices for their sensitivity. We measured noise current (Fig. 5c) and used this, combined with responsivity, area and bandwidth, to determine the specific detectivity (Fig. 5d). Excess noise is responsible for the practical limitations on experimentally achieved values. The SIRD devices are, by a wide margin, the most sensitive photodetectors operating below 5 V compared with any prior solution-processed photoconductor reports, with responsivities that are nearly an order of magnitude higher than previously reported values^39^.

## Discussion

For each of the metal precursors we studied, the minimum gap distance versus the transverse electrode edge distance varies. Platinum electrodes cannot participate in SIRD behaviour; however, electrodes narrow to a single point of contact that shorts with negligible transverse electrode edge. Gold SIRD electrodes, due to fractal nature of electrode growth, narrow to multiple points of minimum gap distance (<50 nm) resulting in moderate transverse electrode edge. Palladium SIRD electrodes narrow to a long edge with minimum gap distance (>50 nm) with the longest transverse electrode edge. It is possible that the enhancement of SIRD photoconductor devices occurs due to a single point of minimum gap distance, but since there are multiple minimum points for Au SIRD electrodes we expect the effect to be distributed over these points.

Another important aspect to consider is field enhancement due to morphology. The fractal morphology of gold SIRD electrodes exhibits sharp edges that can provide significant field enhancement over smooth surfaces with the same minimum gap distance. Sharp-edge field enhancement effects have been studied in analogous electrode morphologies^40^. The distribution of the potential field is shifted such that the multiple minimum gap points produce the strongest electric field and likely contributes to the improved performance at low bias. In addition, SIRD electrodes are three-dimensional in nature at the minimum gap distance, and not restricted to a planar interface to CQD films. Infiltration with CQD films into SIRD electrodes results in high surface area contact at minimum gap distances providing an additional enhancement feature.

SIRD electroplating is a simple and highly scalable method to fabricate robust nano-spaced, electrically independent electrodes. It enables nanoscale-defined devices to be formed from microscale photolithographic-defined templates. It offers material flexibility since various metal precursors could be utilized for SIRD electrode formation. Through experiment and simulation, we found that electroplating kinetics can be exploited to control the degree of self-inhibition and program the resultant nanoscale gap dimensions. Our results confirm that the SIRD electrodes can be applied to forming improved-performance (in this case, lower voltage) devices such as photodetectors. The resultant SIRD-enabled devices demonstrated record performance relative to controls and prior comparable photodetector reports.

## Methods

### SIRD device fabrication

Standard square glass wafers were coated with 5 nm Cr followed by 100 nm of Au. A positive photoresist was spin coated onto the surface and imaged with the SIRD electrode pattern. A 2 μm electrode separation distance was etched with a standard etching process to produce blank unpassivated SIRD electrode chips. Surface of SIRD chips was passivated with a 2 μm layer of SU-8 and imaged with 10-μm apertures on top of the narrow electrode region. SIRD oxide devices were fabricated by sputter coating entire wafer with 50 nm of SiO.

### SIRD electrodeposition

SIRD electrode pairs were connected in parallel in a standard three electrode electrochemical cell consisting of Ag/AgCl reference and Pt counter electrodes. SIRD devices were submersed in plating solution consisting of 2 mM HAuCl_4_ or H_2_PtCl_6_ or PdCl_2_ along with 50 mM of HCl as supporting electrolyte. Simple amperometry was performed to plate SIRD electrode pairs at constant potential in the designated plating solution. Electrical short testing was performed using linear sweep voltammetry between SIRD plated electrodes. SEM analysis was performed using a Hitachi S-3400, and SEM contrast and brightness were adjusted globally to equalize the brightest regions.

### COMSOL simulations

Simulations of electroplating SIRD devices were made using the electrodeposition module in moving mesh mode. Simulation geometry was designed to match experimental devices. An initial concentration of 2 mM, plating overpotential of −0.15 V and diffusion constants of 1.1 × 10^−9^ m^2^ s^−1^ and 2.0 × 10^−9^ m^2^ s^−1^ were utilized for fast and slow simulations from 0 to 5 min. Species charge too was specified to 2 (Pd) and 4 (Pt). Electroplating kinetics of slow and fast conditions were simulated by testing a wide range of exchange current densities from 1 to 500 A m^−2^. Exchange current densities of 5 (Pt) and 150 A m^−2^ (Pd) were found to match that of experimental morphologies.

### Fabrication of CQD photoconductor devices

PbS quantum dots were synthesized according to a previously published method^38^. P-type PbS CQD film was prepared using a dip-coating process. SIRD electrode devices were pre-dipped into 0.05% mercaptopropionic acid in methanol for five seconds, then dried in air for 3 min. The substrate was then dipped into a PbS CQD solution (7.5 mg/ml) in hexane for 30 s, then dried in air for 3 min. The film was consequently dipped into a 0.2% mercaptopropionic acid in methanol solution for 3 sec and dried in air for 1 min. Finally, the film was dipped in methanol for 5 sec and dried in air for 2 min. The cycle was repeated three times for a total thickness of ∼30 nm.

### EQE measurements

The EQE spectrum was obtained using a 400 W xenon lamp and passing the output through a monochromator and order-sorting filters. The collimated beam from the monochromator was mechanically chopped at 80 Hz and measured through a 600-μm aperture using calibrated Newport 818-UV and 818-IR power metres. The current response was measured using a Stanford Research Systems SR830 lock-in amplifier at short-circuit conditions. The accuracy of the EQE measurements was estimated to be 8%.

### Responsivity and irradiance measurements

The responsivity was measured using a 450-nm light-emitting diode (LED) light source incident on the active area of the device, square wave modulated using an Agilent 33220A function generator at 8 Hz. The intensity of the light source was measured using a Newport 818-UV power metre. The optical power impinging on the device active area was approximated by dividing the active area of the device by the light source aperture area and multiplying by the total power measured. The device was biased at 1 V using a Keithley 2400 sourcemeter, while the resulting electrical current was measured using a Stanford Research Systems SR830 lock-in amplifier. Current-irradiance measurements was performed in a similar fashion, with the LED light source powered by a Keithley 2400 sourcemeter. The current through the LED ranges from 0 to 100 mA, providing a range of incident optical power on the device, with the resulting electrical current to be measuring using a second Keithley sourcemeter.

### Noise current and detectivity measurements

Dark current noise through the devices was measured using a Stanford Research Systems SR830 lock-in amplifier. Testing was performed under room temperature in an optically sealed, electrically shielded enclosure on a floating table. The noise current was normalized to the measurement bandwidth and was divided by the responsivity obtained from the same measurement conditions to calculate the NEP. In turn, the detectivity was evaluated as a function of applied bias by dividing the square root of the active area of the photodetector by the NEP. The accuracy of the current–voltage measurements was estimated to be ±7%.

## Author contributions

B.L., S.O.K. and E.H.S. developed the concepts described and designed experiments to be performed; B.L. fabricated SIRD devices, performed characterization experiments and simulations; W.Z. fabricated photodetector SIRD devices and performed photodetector measurements; B.L., W.Z., S.O.K. and E.H.S wrote the manuscript.

## Additional information

**How to cite this article:** Lam, B. *et al.* Programmable definition of nanogap electronic devices using self-inhibited reagent depletion. *Nat. Commun.* 6:6940 doi: 10.1038/ncomms7940 (2015).

## Supplementary Material

Supplementary Information Supplementary Figure 1, Supplementary Methods and Supplementary References
