# Supplementary material for: Programmable definition of nanogap electronic devices using self-inhibited reagent depletion
Source: Nat Commun. 2015 Apr 27;6:6940. doi: 10.1038/ncomms7940 (PMC4423216; doi:10.1038/ncomms7940)
Supplement: Supplementary Information — Supplementary Figure 1, Supplementary Methods and Supplementary References [file ncomms7940-s1.pdf]

Supplementary Figure:

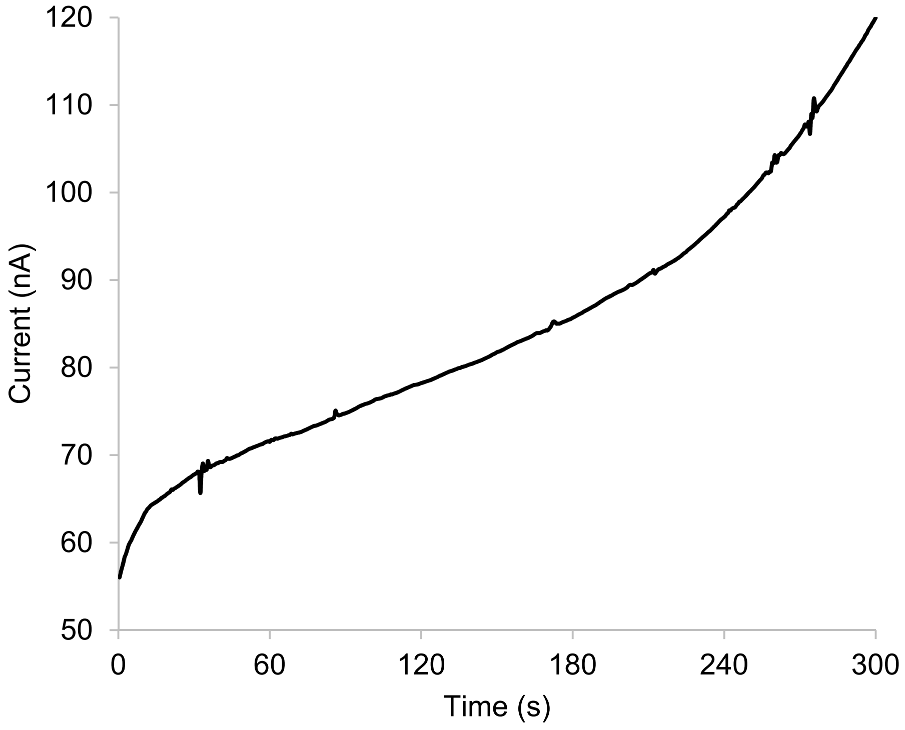

Supplementary Figure 1. Raw electroplating current versus time for Pd for chips consisting of 10 pairs of SIRD electrodes.

Supplementary Methods:

Calculation of diffusion coefficient from experimentally measured values

We estimate that electrode pairs consisting of two half circles with 4 μm radius that are exposed and separated by 2 μm act as a single microelectrode, since both electrodes are electroplated simultaneously at the same potential. We estimate that both electrodes act similar to single circular disk microelectrode embedded in an insulating medium. For a circular disk microelectrode the steady state current (i_{ss}) can be calculated from the following[1],

$$i_{ss} = 4nFDC^{*}r_{o} \qquad (1)$$

Where n is the stoichiometric number of electrons involved, F is Faraday's constant, C^* is the bulk concentration, D the diffusion coefficient and r_o the circular disk microelectrode radius. Shown in Supplementary Figure 1 are electroplating curves for SIRD chips comprising of 10 pairs of electrodes per chip being electroplated simultaneously. The steady state current can be estimated at the time which the plating current becomes linear (~30 sec), since this represents the point at which the growth of the electrode occurs at a constant rate. This indicates an i_{ss} of 68 nA. For our system where $C^* = 2$ mM, $r_o = 4$μm and $n = 2$ we find that $D \approx 1.1 * 10^{-9}$ m^2 s^{-1}.

Supplementary References:

1. Baur, J. E. in *Handb. Electrochem.* 829–49 (2007).
